# Severe Fever with Thrombocytopenia Syndrome in South Korea, 2013-2015

**DOI:** 10.1371/journal.pntd.0005264

**Published:** 2016-12-29

**Authors:** Seong Jin Choi, Sang-Won Park, In-Gyu Bae, Sung-Han Kim, Seong Yeol Ryu, Hyun Ah Kim, Hee-Chang Jang, Jian Hur, Jae-Bum Jun, Younghee Jung, Hyun-Ha Chang, Young Keun Kim, Jongyoun Yi, Kye-Hyung Kim, Jeong-Hwan Hwang, Yeon-Sook Kim, Hye Won Jeong, Kyoung-Ho Song, Wan Beom Park, Eu Suk Kim, Myoung-don Oh, for Korea SFTS Clinical Network

**Affiliations:** 1 Department of Internal Medicine, Seoul National University College of Medicine, Seoul, Republic of Korea; 2 Department of Internal Medicine, Boramae Medical Center, Seoul, Republic of Korea; 3 Department of Internal Medicine, Gyeongsang National University School of Medicine, Jinju, Republic of Korea; 4 Department of Infectious Diseases, Asan Medical Center, University of Ulsan College of Medicine, Seoul, Republic of Korea; 5 Department of Internal Medicine, Keimyung University Dongsan Medical Center, Daegu, Republic of Korea; 6 Department of Infectious Diseases, Chonnam National University Medical School, Gwangju, Republic of Korea; 7 Department of Internal Medicine, Yeungnam University College of Medicine, Daegu, Republic of Korea; 8 Department of Internal Medicine, Ulsan University Hospital, University of Ulsan College of Medicine, Ulsan, Republic of Korea; 9 Department of Internal Medicine, Hallym University Sacred Heart Hospital, Anyang, Republic of Korea; 10 Department of Internal Medicine, Kyungpook National University School of Medicine, Daegu, Republic of Korea; 11 Department of Internal Medicine, Yonsei University Wonju College of Medicine, Wonju, Republic of Korea; 12 Department of Laboratory Medicine, Pusan National University Hospital, Busan, Republic of Korea; 13 Department of Internal Medicine, Pusan National University Hospital, Busan, Republic of Korea; 14 Department of Internal Medicine, Chonbuk National University Medical School, Jeonju, Republic of Korea; 15 Department of Internal Medicine, Chungnam National University School of Medicine, Daejeon, Republic of Korea; 16 Department of Internal Medicine, Chungbuk National University College of Medicine, Cheongju, Republic of Korea; 17 Department of Internal Medicine, Seoul National University Bundang Hospital, Seongnam, Republic of Korea; University of Washington, UNITED STATES

## Abstract

**Background:**

Severe fever with thrombocytopenia syndrome (SFTS) is an emerging infectious disease that was recently identified in China, South Korea and Japan. The objective of the study was to evaluate the epidemiologic and clinical characteristics of SFTS in South Korea.

**Methods/Principal Findings:**

SFTS is a reportable disease in South Korea. We included all SFTS cases reported to the Korea Centers for Disease Control and Prevention (KCDC) from January 2013 to December 2015. Clinical information was gathered by reviewing medical records, and epidemiologic characteristics were analyzed using both KCDC surveillance data and patient medical records. Risk factors for mortality in patients with SFTS were assessed. A total of 172 SFTS cases were reported during the study period. SFTS occurred throughout the country, except in urban areas. Hilly areas in the eastern and southeastern regions and Jeju island (incidence, 1.26 cases /10^5^ person-years) were the main endemic areas. The yearly incidence increased from 36 cases in 2013 to 81 cases in 2015. Most cases occurred from May to October. The overall case fatality ratio was 32.6%. The clinical progression was similar to the 3 phases reported in China: fever, multi-organ dysfunction, and convalescence. Confusion, elevated C-reactive protein, and prolonged activated partial thromboplastin times were associated with mortality in patients with SFTS. Two outbreaks of nosocomial SFTS transmission were observed.

**Conclusions:**

SFTS is an endemic disease in South Korea, with a nationwide distribution and a high case-fatality ratio. Confusion, elevated levels of C-reactive protein, and prolonged activated partial thromboplastin times were associated with mortality in patients with SFTS.

## Author Summary

Severe fever with thrombocytopenia (SFTS) is an emerging infectious disease that was first discovered in China in 2009. Subsequently, SFTS has also been found in South Korea and Japan. Here, we report the epidemiologic and clinical characteristics of 172 confirmed SFTS cases in South Korea that occurred since the first case was reported in 2013. SFTS was prevalent throughout South Korea, except for in urban areas. The incidence was relatively low in the western and southwestern rice field areas and the scarcely populated eastern mountainous area. Hilly areas were the major endemic regions. The incidence was increasing annually, and the case fatality ratio was 32.6%. A mental status of confusion, elevated levels of C-reactive protein, and prolongation of activated partial thromboplastin time were associated with mortality in patients with SFTS. Two outbreaks of nosocomial SFTS transmission were noted.

## Introduction

Severe fever with thrombocytopenia syndrome (SFTS) is an emerging infectious disease that is caused by a novel SFTS virus (SFTSV) which was first reported in China in 2011 [1]. China’s neighboring two countries, South Korea and Japan, have also reported the infection [2, 3]. The virus is transmitted to humans through tick bites, and *Haemaphysalis longicornis* is known to be a main vector [1, 4]. The clinical manifestations of SFTS include fever, myalgia, vomiting, diarrhea, thrombocytopenia and leukopenia. In severe cases, multi-organ dysfunction may occur [5]; the case fatality ratio (CFR) has been reported to be 6.3–30% [6, 7].

In South Korea, the first patient with SFTSV infection was identified in 2012 [2]. Subsequently, an epidemiologic study reported that 35 cases of SFTS, with a CFR of 45.7%, occurred in 2013 [8]. The objective of this study was to characterize the epidemiologic and clinical findings of all SFTS patients since the first case was reported in South Korea.

## Patients and Methods

### The patients

SFTS has been a reportable disease in South Korea since 2013. We included all SFTS cases reported to the Korea Centers for Disease Control and Prevention (KCDC) from 2013 to 2015 [9, 10]. Confirmatory tests of SFTSV infection were performed at KCDC by detecting M segment gene of SFTSV RNA using one-step reverse transcription polymerase chain reaction (RT-PCR) or antibody tests with immunofluorescence assay (IFA) to detect the seroconversion of paired sera for anti-SFTSV immunoglobulin G, as previously described [8, 11].

### Data collection

To collect clinical data, we reviewed the medical records of patients who had available epidemiologic information, clinical manifestations and laboratory findings. Epidemiologic characteristics were also supplemented by reviewing the epidemiology investigation records provided by the KCDC.

Demographic factors, date of onset, history of tick bite, presence of bite wound, and comorbidity were included. History of tick bite was self-reported and collected from medical records. The locations of possible exposure to SFTSV were determined considering the patient’s history of outdoor activities or residential scope in one month prior to the onset of illness [8]. We used the KCDC data for the location of SFTS acquisition. Each case was coded according to its geographic location at the district level and was positioned on a map of South Korea (http://www.gadm.org). The national and regional incidences of SFTS per 100,000 person-years from 2013 to 2015 were calculated using the national census (http://kosis.kr). We divided the clinical course of SFTS into three stages by week, and a comparison of clinical and laboratory features was performed for the fatal and non-fatal cases occurring in each period. Worst values were selected for the data in each patient if there were multiple measurements during the unit period.

### Definition of terms

Meningoencephalitis was defined as a white blood cell count of the cerebrospinal fluid >5 cells/mm^3^. Myocarditis was determined by an abnormal electrocardiography (ECG), serum levels of troponin or creatine phosphokinase (CK) fractions and an echocardiogram. Arrhythmia was defined as either a new onset during the course of illness or a previously undiagnosed new case. Acute kidney injury was defined as serum creatinine levels ≥2 mg/dl and 2 times the baseline levels [5, 12]. The Acute Physiology and Chronic Health Evaluation (APACHE II) score was also calculated [13]. Severe thrombocytopenia was defined as platelet count <50x10^3^/mm^3^ in view of its implication for the critical threshold that a risk of spontaneous bleeding increases [5, 14]. The prolongation of activated partial thromboplastin time (aPTT) was defined as aPTT >60 sec, indicating >50% of upper normal value (reference value of aPTT <40 sec) [5, 15].

### Statistical analysis

Statistical analyses were performed using Pearson’s chi-square test or Fisher’s exact test to analyze the relationships between categorical variables and severity of disease. Two-sample t-tests or Mann-Whitney U-tests were used to compare the continuous variables between fatal and non-fatal cases. Longitudinal analysis for serial clinical feature and laboratory parameters was performed using a multivariable generalized estimating equation with binomial variable and linear mixed model with continuous variable. The risk factors for mortality in patients with SFTS were analyzed by binary logistic regression. *P* values <0.05 were considered statistically significant. Variables having P values <0.05 in the univariate analysis were used for a multivariate stepwise logistic regression analysis. We sought sensitivity, specificity, positive predictive value (PPV), and negative predictive value (NPV) of single variable from 1st week after the onset of illness which was significant in univariate analysis. We also analyzed sensitivity, specificity, and C-statistics of combined variable from 1st week after the onset of illness which were significant in the multivariate analysis (SPSS 22.0; SPSS Inc., Chicago, IL, USA).

### Ethics statement

This study was approved by the institutional review board (IRB) of Boramae Medical Center (#15-2015-123). All the institutions participating in the clinical network also obtained approval from their IRBs. Personal information was de-identified before collection and the anonymized data were processed by different analyzers. All clinical investigations were conducted according to the principles expressed in the Declaration of Helsinki.

## Results

### The study patients

A total of 170 SFTS cases were reported to the KCDC during the study period of 2013–2015 [9]. Of the 170 SFTS cases, 161 were confirmed by RT-PCR and 9 patients by IFA. We also included 2 additional cases that had a negative conventional RT-PCR but a positive real-time RT-PCR. Therefore, a total of 172 cases of SFTS were included in this study.

### Epidemiologic characteristics

**Fig 1:**
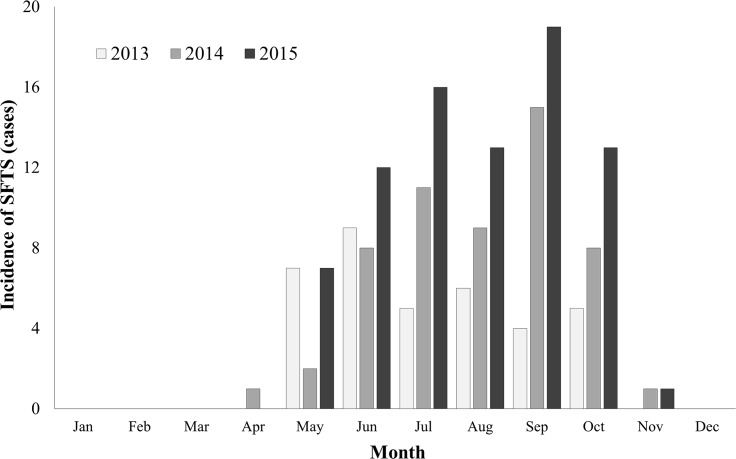
Temporal distribution of 172 patients with severe fever with thrombocytopenia syndrome (SFTS) in South Korea, 2013–2015. The main epidemic period of SFTS was May to October. No cases were reported from December to March, which is the winter season.

**Fig 2:**
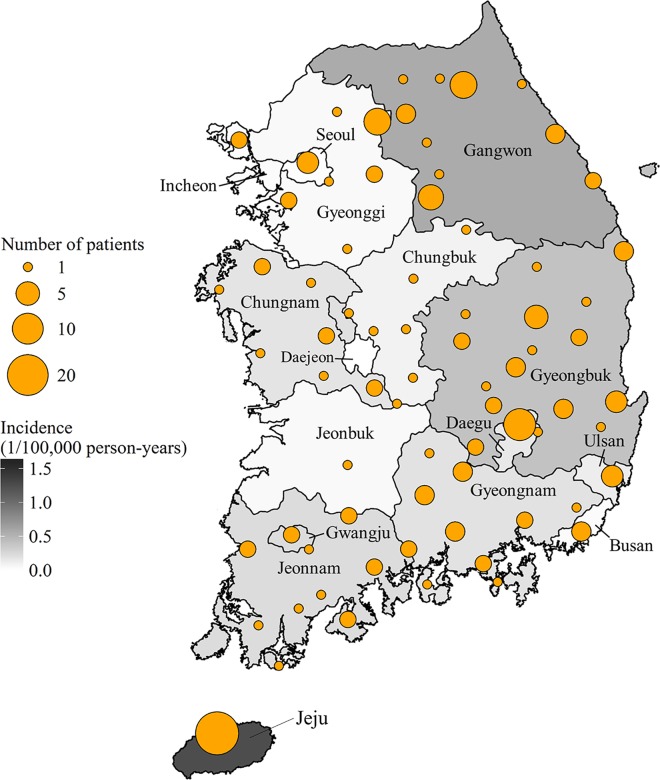
Geographic distribution of 172 patients with severe fever with thrombocytopenia syndrome (SFTS) in South Korea, 2013–2015. SFTS occurred throughout the country with the exception of urban areas. The incidence was relatively low in the western and southwestern rice field areas and the scarcely populated eastern mountainous areas. Hilly areas were the major endemic regions. Orange circles indicate the site of infection, and the size of the circle represents the number of SFTS cases by area. Shading indicates cases per 100,000 persons.

The yearly incidence of SFTS was 36 cases in 2013 (including one case in 2012), 55 cases in 2014, and 81 cases in 2015. The case fatality ratio was 47.2% (17/36) in 2013, 32.7% (18/55) in 2014, and 25.9% (21/81) in 2015, with an overall CFR of 32.6% (56/172). The seasonal distribution of the SFTS cases is shown in Fig 1. Most of the cases occurred between May and October. There were no cases from December to March. The geographical distribution of the SFTS cases is shown in Fig 2. SFTS occurred throughout South Korea, with the exception of urban areas. The incidence was relatively low in the western and southwestern rice field areas and the scarcely populated eastern mountainous areas. Hilly areas were the major endemic regions. The overall incidence was 0.11 cases/10^5^ person-years. Specifically, Jeju province (23 cases, 1.26 cases/10^5^ person-years) showed the highest incidence rate, followed by Gangwon (26 cases, 0.54 cases/10^5^ person-years), Gyeongbuk (33 cases, 0.41 cases/10^5^ person-years), and Jeonnam province (13 cases, 0.23 cases/10^5^ person-years) (Fig 2).

### Clinical characteristics

#### 1. Demographic data

Of the 172 SFTS patients, detailed clinical and laboratory data were not available in 52. Of the 52 patients, 43 patients had been treated by physicians who did not participated in this study, and 9 patients were healthcare workers with nosocomial infection with SFTSV. There were no gender differences between the two groups (120 vs 52 patients), but the median age was different (69.0 vs 61.5 years, p = 0.009). The CFRs of the two groups were 38.3% and 19.2%, respectively (p = 0.014) (S1 Table).

Clinical analyses were performed for the remaining 120 patients. Fifty percent (61/120) of the patients were male. Sixty-four (53.3%) patients did not have any underlying comorbidities; of those who did, hypertension (31.7%), diabetes (18.5%), stroke (4.2%), and asthma (2.5%) were common. Fifty-two (46.4%) patients were farmers. Twenty-six (22.0%) patients recalled a tick bite, and 36 (30.3%) patients had a tick-bite wound at their initial hospital visit (Table 1).

**Table 1:** Baseline characteristics of patients with severe fever with thrombocytopenia syndrome in South Korea, 2013–2015.

		No. (%)			
Characteristics		Non-fatal	Fatal	Total	*P* value
		(n = 74)	(n = 46)	(n = 120)	
Sex					
	Male	42 (56.8)	19 (41.3)	61 (50.8)	0.100
	Female	32 (43.2)	27 (58.7)	59 (49.2)	
Age, years (median; IQR)		66 (52–74)	73.5 (66–79)	69 (59–77)	<0.001
Occupation					
	Farmers	26 (38.2)	26 (59.1)	52 (46.4)	0.031
	Non-farmers	42 (61.7)	18 (40.9)	60 (53.6)	
Tick bite					
	Memory of tick bite	15 (20.8)	11 (23.9)	26 (22.0)	0.694
	Presence of bite wound	21 (28.8)	15 (32.6)	36 (30.3)	0.657
Complications					
	Meningoencephalitis	11 (15.1)	5 (11.6)	16 (13.8)	0.604
	Mechanical ventilation	6 (8.1)	31 (68.9)	37 (31.1)	<0.001
	Myocarditis	2 (2.7)	3 (6.7)	5 (4.2)	0.365
	Arrhythmia	3 (4.1)	11 (24.4)	14 (11.8)	0.002
	Acute kidney injury	3 (4.1)	14 (43.8)	17 (14.2)	<0.001
	Other^a^	1 (1.4)	5 (10.9)	6 (5.0)	0.030
Comorbidity					
	DM	12 (16.2)	10 (22.2)	22 (18.5)	0.413
	HTN	19 (25.7)	19 (41.3)	38 (31.7)	0.074
	CVA	2 (2.7)	3 (6.5)	5 (4.2)	0.370
	CHF	1 (1.4)	1 (2.2)	2 (1.7)	1.000
	CLD	1 (1.4)	1 (2.2)	2 (1.7)	1.000
	CKD	0 (0)	0 (0)	0 (0)	
	Asthma	2 (2.7)	1 (2.2)	3 (2.5)	1.000
	COPD	1 (1.4)	1 (2.2)	2 (1.7)	1.000
	Solid tumor	2 (2.7)	1 (2.2)	3 (2.5)	1.000
	No comorbidity	42 (56.8)	22 (47.8)	64 (53.3)	0.340
Onset of illness to admission		4 (3–6)	4 (2–5)	4 (3–6)	0.500
(median days, IQR)					

^a^Other complications include rhabdomyolysis and subdural hemorrhage.

IQR: interquartile range, DM: diabetes mellitus, HTN: hypertension, CVA: cerebrovascular accident, CHF: congestive heart failure, CLD: chronic liver disease, CKD: chronic kidney diseases, COPD: chronic obstructive pulmonary disease.

#### 2. Clinical presentation and progression

The median time from the onset of illness to the hospital visit was 4 days (interquartile range [IQR], 3–6 days). The major clinical manifestations exhibited in the 1^st^ week of illness were fever (93.5%), myalgia (63.3%), diarrhea (54.3%), anorexia (53.8%), nausea (37.4%), headache (34.1%), and confusion (25.6%) (S2 Table). Lymphadenopathy was identified in 9 (11.5%) patients, and inguinal lymph node involvement was common. Systemic symptoms including fever occurred in 95.7% of the patients, gastrointestinal symptoms in 78%, central nervous symptoms in 66.3%, respiratory and cardiovascular symptoms in 41.8%, and hemorrhagic signs in 21.5% of the 92 patients (S3 Table). Fever persisted for a median of 8 days (IQR, 6–11 days), gastrointestinal symptoms for 10 days (IQR, 8–14 days), and central nervous symptoms for 8 days (IQR, 7–17 days).

**Fig 3:**
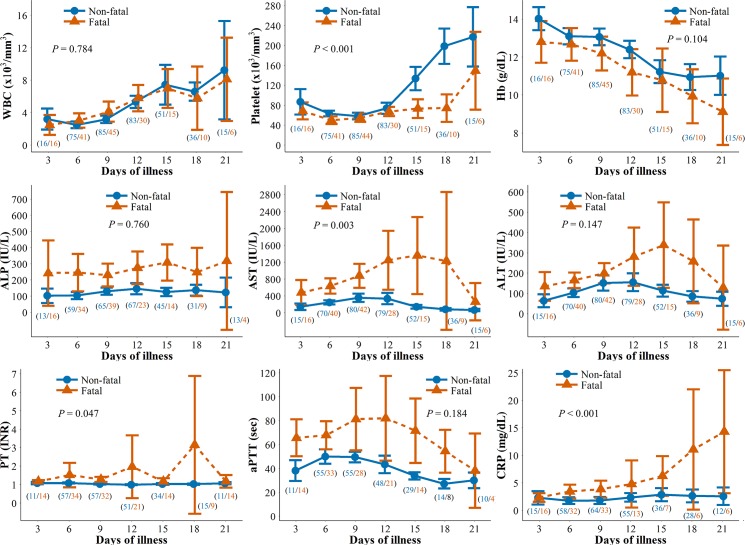
Changes in laboratory parameters over time by intervals of 3 days in 120 patients with severe fever with thrombocytopenia syndrome. The Y-axis values denote the mean and 95% confidence intervals. Blue and red lines indicate the non-fatal and fatal groups, respectively. The number in parenthesis shows non-fatal (blue) and fatal (red) patients included in the analysis at each time point. P values show the statistical significance of variables at each time point over 3 weeks between the two groups. WBC, white blood cell; Hb, hemoglobin; ALP, serum alkaline phosphatase; AST, serum aspartate aminotransferase; ALT, serum alanine aminotransferase; PT, prothrombin time; aPTT, activated partial thromboplastin time; CRP, C-reactive protein.

The changes in laboratory parameters over time are shown in Fig 3 & S4 Table. During the 1^st^ week of illness, thrombocytopenia (96.7%) and leukopenia (91.3%) were most common. Elevated levels of serum aspartate aminotransferase (AST) (95.7%), alanine aminotransferase (ALT) (90.2%), lactate dehydrogenase (LDH) (94.0%), creatine kinase (CK) (88.5%), ferritin (66.7%), and C-reactive protein (CRP) (21.3%) were also observed. Hypoalbuminemia (<3.5 g/dL) and hyponatremia (<135 mmol/L) were observed in 75.3% and 48.3% of patients, respectively. The leukocyte count decreased to a minimum in a median of 7 days (IQR, 5–8 days) and returned to a normal range in the 2^nd^ and 3^rd^ week of survived patients. The platelet count decreased to a minimum in a median of 8 days (IQR, 5–10 days) and reverted to a normal range in the 3^rd^ week in non-fatal cases. However, thrombocytopenia persisted in 76.9% (10/13) of patients in the fatal group, and 3 patients in the fatal group recovered from thrombocytopenia at the end of the 3^rd^ week. AST, ALT, and LDH peaked in a median of 8 days (IQR, 6–10 days) and then gradually returned normal. An increase in CRP was observed in 66.7% (6/9) of fatal patients in the 3^rd^ week (Fig 3 & S4 Table).

### Risk factors for mortality

Of the 120 patients, 37 (31.1%) were placed on mechanical ventilation. Acute renal failure (14.2%), meningoencephalitis (13.8%), new-onset arrhythmia (11.8%), and myocarditis (4.2%) were common complications during the hospital course (Table 1). Forty-six patients died, resulting in a CFR of 38.3%. The median time from onset of illness to death was 9.5 days (IQR, 7–15 days); 41.3% (19/46) of the non-surviving patients were male, and the median age was 73.5 years (IQR, 66–79 years). Of the surviving patients, 56.8% (42/74) were male, and the median age was 66 years (IQR, 52–74 years), which was lower than that of the fatal group (p < 0.001) (Table 1). The median time from the onset of illness to hospital discharge was 16 days (IQR, 13–23 days) in the non-fatal group.

Among the clinical parameters in the 1^st^ week, dyspnea, gastrointestinal bleeding, and confusion were associated with death (Table 2). In terms of the categorical clinical features, the frequency of central nervous symptoms was higher in the fatal group (p = 0.025) (S3 Table). In the univariate analysis of laboratory parameters in week 1, severe thrombocytopenia (<50×10^3^/mm^3^) was more common in the fatal group (43.1% vs 70.6%, *p* = 0.012). Anemia, increases in serum alkaline phosphatase, AST (>400 IU/L), ALT (> 200 IU/L) and CRP (mg/dL), and prolongation of prothrombin time (PT) (INR ≥1.3) and aPTT (>60 sec) were associated with death (Table 2).

**Table 2:** Risk factors of mortality in the 1^st^ week after the onset of illness in SFTS.

	Univariate analysis			Multivariate analysis		
Variable	OR	95% CI	*P* value	Adjusted OR	95% CI	*P* value
Sex (female)	2.53	1.08–6.18	**0.037**	3.60	1.01–15.14	0.052
Age	4.00	1.57–11.28	**0.005**			
Dyspnea	5.26	1.83–16.75	**0.003**			
GI bleeding	5.89	1.27–52.04	**0.037**			
Confusion	9.03	3.19–28.79	**<0.001**	8.48	2.16–40.90	**0.005**
Leukopenia (<4,000/mm^3^)	4.52	0.76–86.69	0.167			
Anemia (<11 g/dL)	3.86	1.42–11.12	**0.009**			
Thrombocytopenia (<50×10^3^/mm^3^)	3.16	1.31–8.08	**0.012**	3.13	0.87–12.78	0.090
Elevated ALP (>120 IU/L)	3.29	1.31–8.59	**0.013**			
Elevated AST (≥400 IU/L)	3.29	1.24–9.91	**0.023**			
Elevated ALT (≥200 IU/L)	3.81	1.45–10.55	**0.008**			
Elevated CRP (mg/dL)	1.20	1.04–1.46	**0.025**	1.23	1.03–1.54	**0.034**
PT prolongation (INR ≥1.3)	7.97	2.24–37.79	**0.003**			
aPTT prolongation (≥60 sec)	4.29	1.70–11.38	**0.003**	4.53	1.33–16.57	**0.017**

GI: gastrointestinal, ALP: alkaline phosphatase, AST: aspartate aminotransferase, ALT: alanine aminotransferase, PT: prothrombin time, aPTT: activated partial thromboplastin time, CRP: C-reactive protein, OR: odds ratio

APACHE II scores were higher in the fatal group than in the non-fatal group (15 vs 23, *p* <0.001).

Changes of clinical and laboratory variables over 3 weeks between the two groups were significantly different for the variables of confusion (p < 0.001), respiratory and cardiovascular symptoms (p = 0.022), platelet (p < 0.001), AST (p = 0.005), CRP (p < 0.001), serum creatinine (p < 0.001), and LDH (p < 0.001) (S2, S3 and S4 Tables & Fig 3). In the multivariate regression analysis, a mental state of confusion, elevated levels of CRP, and the prolongation of aPTT were associated with mortality (Table 2). The single variable for the highest value of sensitivity (leukopenia, 97.1%), specificity (prolongation of PT, 94.3%), PPV (prolongation of PT, 78.6%), and NPV (elevated ALT, 80.0%) was not uniform (S5 Table). With combined variables, ‘confusion + aPTT >60 sec’ showed the maximal C-statistics value of 0.786 (95% confidence interval, 0.625–0.948) (S6 Table).

## Discussion

This study showed that SFTS occurred throughout South Korea. The overall incidence of SFTS in South Korea was 0.11 cases per 10^5^ person-years, which was lower than that in China (0.12–0.73 cases per 10^5^ person-years) [16]. The median age of 69.0 years in our patients is higher than that in China, 57.6 years [17], which reflects the difference of aging populations between two countries, especially in rural areas. We also showed that summer was a period of peak transmission. A previous study suggested that *H*. *longicornis* was the predominant species (90.8%) and was widely distributed in a nationwide surveillance of ticks [18]. Although *H*. *longicornis* is the major vector, other tick species such as *Haemaphysalis flava*, *Amblyomma testudinarium* and *Ixodes nipponensis* have also been reported to carry SFTSV in South Korea [19, 20]. The peak transmission in summertime may be attributable to the seasonal life cycle of *H*. *longicornis* [18, 21] and the increased rates of outdoor activity during this season. The western and southwestern areas of South Korea showed a relatively low incidence of SFTS, which might be related to the low SFTSV infection rate in ticks [18]. This is evidenced by the contrasting finding of a high incidence of tsutsugamushi in the same area, which is closely related to people’s outdoor activity and is one of the most common notifiable infectious diseases in South Korea [22, 23]. The main epidemic season of tsutsugamushi in South Korea is autumn, when chigger mites begin sucking blood from mammals to meet the developmental needs of their life cycle. Several SFTS patients were found to be infected in urban areas. However, 4 cases in Seoul and 5 cases in Wonju were from nosocomial outbreaks. All other cases reported in urban areas were from major cities such as Daegu, Ulsan, Busan and Gwangju, and these cases occurred in the rural suburbs of the city border. Therefore, no endemic cases in inner city were found in South Korea.

The overall CFR in our study was 32.6% which was compared with 12.2% reported recently in China [6]. Although the CFRs of consecutive three years has been decreasing, the CFRs may still be exaggerated. Since SFTS is a newly emerging infectious disease in South Korea, more inclusive screening criteria and education will continue to find more patients with milder presentation. But the patients with mild presentations may have so short or no viremic period that real-time RT-PCR for serum cannot prove the presence of SFTSV effectively. The easy accessibility to well-performing serologic method is needed to help the diagnosis for such patients group. The annual increase of SFTS incidence may be interpreted as a result of increased surveillance or awareness otherwise not detected in the past. We need to follow the trend of SFTS incidence further.

The clinical course of our patients was consistent with the 3 previously reported clinical stages of fever, multi-organ dysfunction and convalescence [5]. Our study identified fever, thrombocytopenia and leukopenia in more than 90% of the patients in the 1^st^ stage, and high fever lasted for a median of 6–11 days. Chinese data showed a high-fever period of 5–11 days [7]. In the 2^nd^ stage, biomarkers including AST, ALT and aPTT were elevated to maximum levels. However, there were notable differences in these values between the fatal and non-fatal groups (Fig 3). During the convalescence stage, the clinical symptoms of SFTS patients began to resolve from 8 to 11 days after the onset of illness, and the laboratory parameters gradually returned to their normal ranges. In China, the convalescence stage began approximately 11–19 days after disease onset, and the biochemical measurements returned to normal within approximately 3–4 weeks in survivors [7]. The overall types of clinical parameters and their changes over time were similar in South Korea and China.

In our study, the median time from the onset of illness to death was 9.5 days. The levels of important biomarkers including hemoglobin, platelets, PT, aPTT, serum ALP, AST, ALT and CRP and clinical parameters such as age, dyspnea, gastrointestinal bleeding and confusion all showed significant differences between the fatal and non-fatal groups. Previous studies found that age, neurologic manifestations, hemorrhagic signs, thrombocytopenia, and elevations of AST, CK or LDH were related to mortality in patients with SFTS in univariate analyses [5, 24]. Neurologic manifestations, thrombocytopenia, prolongation of aPTT, hypoalbuminemia and hyponatremia were significant prognostic factors in multivariate analyses [6, 21, 25]. In our study, confusion, elevated levels of CRP, and prolonged aPTT were associated with the death of patients with SFTS in the multivariate analysis. Overall, mental status and hemorrhagic tendency seemed to be the predominant factors closely related to prognosis. We sought the sensitivity/specificity and PPV/NPV for single or combined variables which were significant in the univariate and multivariate analysis. Confusion and aPTT were important predictors for death. Elevated CRP as either continuous or categorical (>3 mg/dL) variable was one of significant predictors for death from several analyses in our study. Secondary infections like pneumonia or catheter related bloodstream infection might complicate the fatal patients. 31.1% of patients underwent mechanical ventilation during the hospital course. But we couldn’t collect precise data for the infectious complications.

Two hospital outbreaks of SFTS occurred, and 9 healthcare workers (HCWs) were infected [11, 26]. In one hospital, 4 of the 27 HCWs who had contacted with an index patient during cardiopulmonary resuscitation were diagnosed with SFTS via seroconversion [11]. In another hospital, 5 of the 27 HCWs who had been exposed to blood and body fluids were also diagnosed with SFTS via seroconversion [26]. All of the healthcare workers had mild or asymptomatic infections. Nosocomial outbreaks were also reported in China, and not only doctors and nurses but also family members and mortuary beauticians were infected. Therefore, standard precautions should be strictly implemented when caring for suspected SFTS patients [27–29].

Several diseases should be mentioned in the differential diagnosis in South Korea. Human granulocytic anaplasmosis (HGA), which is a tick-borne disease, has a similar clinical presentation including fever, thrombocytopenia and leukopenia. The first human case of HGA in South Korea was reported in 2014 [30]. A serologic survey of the blood samples submitted for SFTS tests showed a 2.2% positivity for anaplasmosis [31]. *A*. *phagocytophilum* has been detected in *H*. *longicornis*, *I*. *nipponensis* and *I*. *persulcatus* ticks [32, 33]. Human monocytotrophic ehrlichiosis also has a similar clinical presentation. Ehrlichiosis was reported in an active duty soldier [34], and its causative agent, *Ehrlichia chaffeensis*, was identified in ticks in South Korea [32]. Hemophagocytic lymphohistiocytosis (HLH) is an aggressive and life-threatening disease which is triggered commonly by infection. Its major diagnostic criteria include fever (≥38.5°C), thrombocytopenia, neutropenia, hyperferritinemia (>3,000 ng/mL), CNS symptoms, hepatitis, coagulopathy and hemophagocytosis in bone marrow [35]. These findings were not uncommon in our data (S3 & S4 Tables). Although we did not observe bone marrow findings, hemophagocytosis in the bone marrow of SFTS patients has been reported as one of the key findings [36–38]. As a cause of secondary HLH, SFTS needs to be considered in endemic areas.

Specific antiviral therapies are urgently needed considering the high fatality and widespread prevalence of SFTS in northeast Asian regions. This study did not analyze the effects of antiviral treatment for SFTS because of the limited number of cases. Several regimens have been tested based on individual physician’s decisions, including a combination of plasma exchange and ribavirin administration [39], plasma exchange followed by convalescent plasma therapy [40], and combination of intravenous immunoglobulin and corticosteroid [41]. Ribavirin showed *in vitro* antiviral effects against SFTSV in a dose-dependent manner [42]. However, a clinical study found that ribavirin monotherapy was not effective [43]. Recent studies suggest that favipiravir and a combination of ribavirin and interferon may be effective in treating SFTS infection [44, 45].

This study has some limitations. As the study data were retrospectively collected from many study sites, the clinical variables affecting the risk factors for death could be incompletely assessed. But most of the SFTS patients were referred to infectious disease physicians, and usually the patients had intensive medical scrutiny with frequent laboratory evaluations. We presented the epidemiologic features of all 172 patients who had SFTS in a period of 3 years. However, in the clinical analysis, we excluded 52 patients. The two groups (120 vs 52 patients) showed significant differences in age distribution and CFR. This is because the group of 52 patients included the nosocomial outbreak cases, which occurred in younger ages and had a mild presentation.

In conclusion, SFTS is a prevalent endemic disease in South Korea that has a high case-fatality ratio. The clinical manifestations were similar to those reported in China. Confusion, elevated levels of C-reactive protein, and prolonged activated partial thromboplastin times were associated with death in patients with SFTS. The development of effective therapeutics for SFTSV infection is urgently needed.

## Supporting Information

S1 Table Demographic differences between SFTS patients with (A) and without (B) detailed clinical and laboratory data.
(DOCX)

S2 Table Changes in clinical manifestations of patients with SFTS over time after the onset of illness. (DOCX)

S3 Table Changes in categorical clinical features of patients with SFTS over time after the onset of illness. (DOCX)

S4 Table Changes in laboratory parameters of patients with SFTS over three weeks in fatal and non-fatal groups. (DOCX)

S5 Table Sensitivity, specificity, positive predictive value (PPV), and negative predictive value (NPV) of single variable from 1^st^ week after the onset of illness which was significant in the univariate analysis. (DOCX)

S6 Table Sensitivity, specificity, C-statistics and 95% confidence interval (CI) of combined two variables from 1st week after the onset of illness which were significant in the multivariate analysis. (DOCX)
