# Supplementary material for: Severe Fever with Thrombocytopenia Syndrome in South Korea, 2013-2015
Source: PLoS Negl Trop Dis. 2016 Dec 29;10(12):e0005264. doi: 10.1371/journal.pntd.0005264 (PMC5226827; doi:10.1371/journal.pntd.0005264)
Supplement: S1 Table — Demographic differences between SFTS patients with (A) and without (B) detailed clinical and laboratory data. (DOCX) [file pntd.0005264.s001.docx]

| **Supplementary table 1.** Demographic difference between SFTS patients with (A) and without (B) detailed clinical and laboratory data | | | | | | |
| --- | --- | --- | --- | --- | --- | --- |
| Demographic characteristics | | | A, n=120 | B, n=52 | Total, n=172 | *P* value |
| Sex, n (%) | |  |  |  |  |  |
|  | Male | | 61 (70.9) | 25 (20.1) | 86 (50.0) | 0.868 |
|  | Female | | 59 (68.6) | 27 (31.4) | 86 (50.0) |  |
| Age, median (IQR) | |  | 69 (59-77.25) | 61.5 (51.75-72) | 67.5 (57-76) | **0.009** |
| Fatality, n (%) | |  | 46 (38.3) | 10 (19.2) | 56 (32.6) | **0.014** |
