# Supplementary material for: Severe Fever with Thrombocytopenia Syndrome in South Korea, 2013-2015
Source: PLoS Negl Trop Dis. 2016 Dec 29;10(12):e0005264. doi: 10.1371/journal.pntd.0005264 (PMC5226827; doi:10.1371/journal.pntd.0005264)
Supplement: S2 Table — (DOCX) [file pntd.0005264.s002.docx]

| **Supplementary table 2.** Changes in clinical manifestations of patients with SFTS over time after the onset of illness | | | | | | | | | | | |
| --- | --- | --- | --- | --- | --- | --- | --- | --- | --- | --- | --- |
|  |  | 1^st^ week, n^*^ (%) | |  | 2^nd^ week, n^*^ (%) | |  | 3^rd^ week, n^*^ (%) | |  | *P* value^**^ |
| Variable | | Non-fatal | Fatal |  | Non-fatal | Fatal |  | Non-fatal | Fatal |  |  |
| Systemic symptoms | |  |  |  |  |  |  |  |  |  |  |
|  | Fever | 54 (93.1) | 32 (94.1) |  | 35 (50.0) | 25 (78.1) |  | 6 (12.8) | 5 (38.5) |  | 0.575 |
|  | Myalgia | 39 (68.4) | 18 (54.5) |  | 18 (26.1) | 13 (44.8) |  | 6 (13.0) | 2 (22.2) |  | 0.231 |
|  | Arthralgia | 6 (10.5) | 1 (3.0) |  | 6 (9.1) | 1 (3.4) |  | 2 (4.7) | 0 |  | 0.779 |
|  | Back pain | 7 (12.3) | 3 (9.1) |  | 4 (6.1) | 4 (13.8) |  | 1 (2.3) | 0 |  | 0.397 |
|  | Sore throat | 6 (10.5) | 1 (2.9) |  | 8 (12.1) | 1 (3.6) |  | 2 (4.5) | 0 |  | 0.942 |
| Respiratory and Cardiovascular symptoms | |  |  |  |  |  |  |  |  |  |  |
|  | Cough | 10 (17.5) | 3 (8.8) |  | 10 (14.9) | 5 (17.9) |  | 3 (6.8) | 0 |  | 0.655 |
|  | Sputum | 12 (21.1) | 8 (23.5) |  | 18 (26.5) | 12 (42.9) |  | 7 (16.3) | 0 |  | 0.396 |
|  | Dyspnea | 6 (10.5) | 13 (38.2) |  | 10 (14.9) | 16 (53.3) |  | 3 (7.0) | 2 (22.2) |  | 0.078 |
|  | Chest pain | 4 (7.0) | 1 (2.9) |  | 0 | 0 |  | 0 | 0 |  | 1.000 |
| Gastrointestinal symptoms | |  |  |  |  |  |  |  |  |  |  |
|  | Anorexia | 31 (54.4) | 18 (52.9) |  | 26 (39.4) | 9 (32.1) |  | 10 (22.7) | 1 (12.5) |  | 0.090 |
|  | Nausea | 25 (43.9) | 9 (26.5) |  | 15 (22.7) | 5 (17.9) |  | 2 (4.5) | 0 |  | 0.604 |
|  | Vomiting | 15 (26.3) | 7 (20.6) |  | 5 (7.5) | 3 (10.7) |  | 2 (4.5) | 0 |  | 0.745 |
|  | Diarrhea | 31 (53.4) | 19 (55.9) |  | 23 (33.3) | 8 (28.6) |  | 4 (8.7) | 1 (11.1) |  | 0.735 |
|  | Abdominal pain | 12 (21.1) | 8 (23.5) |  | 8 (11.9) | 5 (17.2) |  | 2 (4.5) | 0 |  | 0.920 |
| Hemorrhagic signs | |  |  |  |  |  |  |  |  |  |  |
|  | Epistaxis | 1 (1.8) | 1 (2.9) |  | 0 | 1 (3.3) |  | 1 (2.3) | 1 (9.1) |  | 1.000 |
|  | Gingival bleeding | 3 (5.3) | 4 (11.8) |  | 3 (4.5) | 3 (9.7) |  | 0 | 1 (8.3) |  | 0.306 |
|  | Hemoptysis | 1 (1.8) | 2 (5.9) |  | 4 (6.0) | 3 (10) |  | 0 | 0 |  | 0.986 |
|  | Gastrointestinal bleeding | 2 (3.5) | 6 (17.6) |  | 5 (7.5) | 5 (16.7) |  | 1 (2.3) | 1 (9.1) |  | 0.465 |
|  | Hematuria | 1 (1.8) | 2 (5.9) |  | 2 (3) | 3 (10) |  | 0 | 0 |  | 0.899 |
|  | Purpura | 3 (5.3) | 3 (8.8) |  | 2 (3) | 6 (20) |  | 0 | 1 (9.1) |  | 0.067 |
|  | Petechiae | 3 (5.3) | 5 (14.7) |  | 4 (6.0) | 7 (22.6) |  | 2 (4.84 | 3 (25) |  | 0.208 |
| CNS symptoms | |  |  |  |  |  |  |  |  |  |  |
|  | Headache | 20 (35.1) | 11 (32.4) |  | 16 (23.9) | 3 (10.7) |  | 4 (9.3) | 1 (12.5) |  | 0.966 |
|  | Dizziness | 13 (22.8) | 7 (21.2) |  | 6 (9.1) | 0 |  | 2 (4.7) | 0 |  | 0.575 |
|  | Seizure | 4 (7) | 4 (11.8) |  | 6 (9.1) | 8 (26.7) |  | 0 | 2 (18.2) |  | 0.059 |
|  | Confusion | 6 (10.5) | 17 (51.5) |  | 22 (31) | 26 (86.7) |  | 7 (15.2) | 10 (90.9) |  | **< 0.001** |
| Physical findings | |  |  |  |  |  |  |  |  |  |  |
|  | Conjunctival injection | 3 (5.5) | 3 (9.4) |  | 2 (3.2) | 3 (11.1) |  | 0 | 1 (10) |  | 0.144 |
|  | Lymphadenopathy | 7 (12.7) | 3 (9.4) |  | 6 (9.4) | 3 (10.7) |  | 4 (10.3) | 2 (18.2) |  | 0.364 |
| ^*^Due to the odd number of variables in a given time point, the numerator of each variable differs by several cases.  ^**^*P* values show the statistical significance of variables over 3 weeks between non-fatal and fatal groups.  CNS: central nervous system | | | | | | | | | | | |
