# Supplementary material for: Severe Fever with Thrombocytopenia Syndrome in South Korea, 2013-2015
Source: PLoS Negl Trop Dis. 2016 Dec 29;10(12):e0005264. doi: 10.1371/journal.pntd.0005264 (PMC5226827; doi:10.1371/journal.pntd.0005264)
Supplement: S3 Table — (DOCX) [file pntd.0005264.s003.docx]

| **Supplementary table 3.** Changes in categorical clinical features of patients with SFTS over time after the onset of illness | | | | | | | | | | |
| --- | --- | --- | --- | --- | --- | --- | --- | --- | --- | --- |
|  | 1^st^ week, n^*^ (%) | |  | 2^nd^ week, n^*^ (%) | |  | 3^rd^ week, n^*^ (%) | |  | *P* value^**^ |
| Variable | Non-fatal | Fatal |  | Non-fatal | Fatal |  | Non-fatal | Fatal |  |  |
| Systemic symptoms | 56 (96.6) | 32 (94.1) |  | 44 (62.9) | 26 (83.9) |  | 12 (27.3) | 5 (50.0) |  | 0.741 |
| Respiratory and cardiovascular symptoms | 22 (38.6) | 16 (47.1) |  | 20 (30.3) | 18 (64.3) |  | 7 (16.7) | 1 (12.5) |  | **0.022** |
| Gastrointestinal symptoms | 44 (77.2) | 27 (79.4) |  | 41 (62.1) | 15 (53.6) |  | 14 (31.8) | 2 (25.0) |  | 1.000 |
| Hemorrhagic symptoms | 9 (15.5) | 11 (31.4) |  | 13 (18.1) | 12 (27.3) |  | 4 (5.6) | 2 (4.3) |  | 0.766 |
| CNS symptoms | 33 (57.9) | 26 (81.3) |  | 32 (47.8) | 24 (88.9) |  | 11 (26.2) | 6 (85.7) |  | 1.000 |
| ^*^Due to the odd number of variables in a given time point, the numerator of each variable differs by several cases.  ^**^*P* values show the statistical significance of variables over 3 weeks between non-fatal and fatal groups.  CNS: central nervous system | | | | | | | | | | |
