# Supplementary material for: Severe Fever with Thrombocytopenia Syndrome in South Korea, 2013-2015
Source: PLoS Negl Trop Dis. 2016 Dec 29;10(12):e0005264. doi: 10.1371/journal.pntd.0005264 (PMC5226827; doi:10.1371/journal.pntd.0005264)
Supplement: S4 Table — (DOCX) [file pntd.0005264.s004.docx]

| **Supplementary table 4.** Changes in laboratory parameters of patients with SFTS over three weeks in fatal and non-fatal groups | | | | | | | | | |
| --- | --- | --- | --- | --- | --- | --- | --- | --- | --- |
| Variable | Non-fatal (Median (95% CI)) | | |  | Fatal (Median (95% CI)) | | |  | *P* value^**^ |
|  | 1st week (n=58^*^) | 2nd week (n=70^*^) | 3rd week (n=43^*^) |  | 1st week (n=34^*^) | 2nd week (n=33^*^) | 3rd week (n=13^*^) |  |  |
| WBC (/mm^3^) | 1800 (1760-2880) | 2600 (2670-3610) | 5600 (5000-9230) |  | 1600 (1230-2850) | 3200 (2990-5590) | 4820 (3030-8430) |  | 0.162 |
| Platelet (x10^3^/mm^3^) | 53 (48.1-63) | 47.5 (47.3-64) | 185 (164.9-227.4) |  | 41 (36.2-47.3) | 43 (42-63.1) | 54 (46.6-84.4) |  | **< 0.001** |
| Hb (g/dL) | 12.8 (12.3-13.3) | 11.7 (10.9-11.9) | 10.9 (10.3-11.4) |  | 12 (10.8-12.7) | 10.2 (9.0-11.2) | 8.7 (8.2-10.6) |  | 0.478 |
| Albumin (g/dL) | 3.1 (3.1-3.4) | 2.7 (2.7-3) | 3.1 (2.8-3.2) |  | 2.8 (2.5-3) | 2.5 (2.1-2.5) | 2.3 (2.1-2.6) |  | 0.170 |
| ALP (IU/L) | 81 (93-143) | 109 (118-182) | 98 (96-165) |  | 123 (128-399) | 220 (223-423) | 180 (138-458) |  | 0.454 |
| AST (IU/L) | 270 (236-353) | 245 (271-562) | 49 (53-100) |  | 784 (616-1036) | 1060 (966-2343) | 190 (69-2503) |  | **0.005** |
| ALT (IU/L) | 94 (97-146) | 114 (137-235) | 56 (61-106) |  | 173 (156-249) | 230 (209-503) | 108 (106-466) |  | 0.270 |
| Creatinine (mg/dL) | 0.94 (0.9-1.19) | 0.78 (0.73-0.87) | 0.6 (0.55-0.92) |  | 1.16 (1.13-1.52) | 2.02 (1.79-2.64) | 1.3 (0.81-3.15) |  | **< 0.001** |
| Sodium (mmol/L) | 135 (133.6-136) | 136 (133.9-137.3) | 140 (138.3-140.4) |  | 135 (132.5-135.7) | 138 (135.1-139.2) | 136 (133.2-141.7) |  | 0.159 |
| Potassium (mmol/L) | 3.7 (3.56-3.77) | 3.6 (3.47-3.72) | 3.88 (3.65-3.95) |  | 3.8 (3.69-4.12) | 3.7 (3.54-4.14) | 4.1 (3.67-4.42) |  | 0.979 |
| PT-INR (INR) | 1.1 (1.08-1.14) | 1.02 (1.06-1.08) | 1.05 (1.00-1.08) |  | 1.17 (0.87-2.20) | 1.31 (0.69-3.38) | 1.24 (-0.03-5.36) |  | 0.236 |
| aPTT (sec) | 48 (46-58) | 44 (42-55) | 30 (26-33) |  | 61 (58-103) | 81 (66-127) | 48 (38-72) |  | 0.285 |
| LDH (IU/L) | 895 (848-1418) | 1221 (1199-2304) | 502 (480-888) |  | 2151 (1900-3102) | 4282 (3815-6816) | 1256 (494-3855) |  | **< 0.001** |
| CK (IU/L) | 1026 (1065-2480) | 964 (1109-2908) | 316 (-183-2287) |  | 1537 (1596-6975) | 3516 (2651-7506) | 505 (-3019-12470) |  | 0.119 |
| CRP (mg/dL) | 0.52 (0.74-2.12) | 0.56 (0.93-2.58) | 0.47 (1.10-3.85) |  | 2.26 (1.84-4.90) | 2.22 (1.81-6.66) | 9.5 (4.10-19.83) |  | **< 0.001** |
| ESR (mm/h) | 6.5 (5.2-9.9) | 9 (8.0-28.8) | 35 (25.5-58.3) |  | 9 (7.31-23.6) | 3 (2.6-17.6) | 47 (0-96.1) |  | 0.111 |
| Procalcitonin (ng/mL) | 0.14 (0.18-0.39) | 0.48 (0.19-0.96) | - |  | 0.72 (0.22-2.39) | 1.7 (0.54-5.08) | 0.5 (-7.76-13.45) |  | 0.083 |
| Ferritin (ng/mL) | 2000 (2630-8649) | 8987 (3148-30986) | 5542 |  | 11450 (1911-27182) | 18569 (7103-32993) | 1570 (333-2737) |  | 0.631 |
| ^*^Total number of patients at each week. Due to the retrospective data collection, the number of patients who performed the laboratory test differs by cases  ^**^*P* values show the statistical significance of variables over 3 weeks between non-fatal and fatal groups.  WBC: white blood cell, Hb: hemoglobin, ALP: alkaline phosphatase, AST: aspartate aminotransferase, ALT: alanine aminotransferase, PT: prothrombin time, INR: international normalized ration, aPTT: activated partial thromboplastin time, CK: creatine kinase, LDH: lactate dehydrogenase, CRP: C-reactive protein | | | | | | | | | |
