# Supplementary material for: Severe Fever with Thrombocytopenia Syndrome in South Korea, 2013-2015
Source: PLoS Negl Trop Dis. 2016 Dec 29;10(12):e0005264. doi: 10.1371/journal.pntd.0005264 (PMC5226827; doi:10.1371/journal.pntd.0005264)
Supplement: S5 Table — (DOCX) [file pntd.0005264.s005.docx]

**Supplementary table 5.** Sensitivity, specificity, positive predictive value (PPV), and negative predictive value (NPV) of single variable from 1^st^ week after the onset of illness which was significant in the univariate analysis

| Variable | Sensitivity | Specificity | PPV | NPV |
| --- | --- | --- | --- | --- |
| Age >65 years | 0.800 | 0.500 | 0.491 | 0.806 |
| Dyspnea | 0.382 | 0.895 | 0.684 | 0.708 |
| GI bleeding | 0.176 | 0.965 | 0.750 | 0.663 |
| Confusion | 0.515 | 0.895 | 0.739 | 0.761 |
| Leukopenia (<4,000/mm^3^) | **0.971** | 0.121 | 0.393 | 0.875 |
| Anemia (<11 g/dL) | 0.382 | 0.862 | 0.619 | 0.704 |
| Thrombocytopenia (<50×10^3^/mm^3^) | 0.706 | 0.569 | 0.490 | 0.767 |
| Elevated ALP (>120 IU/L) | 0.548 | 0.731 | 0.548 | 0.731 |
| Elevated AST (≥400 IU/L) | 0.706 | 0.759 | 0.632 | **0.815** |
| Elevated ALT (≥200 IU/L) | 0.412 | 0.845 | 0.609 | 0.710 |
| Elevated CRP (>3 mg/dL) | 0.375 | 0.877 | 0.632 | 0.714 |
| PT prolongation (INR ≥1.3) | 0.324 | **0.943** | **0.786** | 0.685 |
| aPTT prolongation (≥60 sec) | 0.529 | 0.792 | 0.621 | 0.724 |

GI: gastrointestinal, ALP: alkaline phosphatase, AST: aspartate aminotransferase, ALT: alanine aminotransferase, PT: prothrombin time,

aPTT: activated partial thromboplastin time, CRP: C-reactive protein
