# Supplementary material for: Severe Fever with Thrombocytopenia Syndrome in South Korea, 2013-2015
Source: PLoS Negl Trop Dis. 2016 Dec 29;10(12):e0005264. doi: 10.1371/journal.pntd.0005264 (PMC5226827; doi:10.1371/journal.pntd.0005264)
Supplement: S6 Table — (DOCX) [file pntd.0005264.s006.docx]

**Supplementary table 6.** Sensitivity, specificity, C-statistics and 95% confidence interval (CI) of combined two variables from 1^st^ week after the onset of illness which were significant in the multivariate analysis

| Combined variable | | Sensitivity | Specificity | C-statistic | 95% CI |
| --- | --- | --- | --- | --- | --- |
| Sex, female | Confusion | 0.533 | **0.913** | 0.755 | 0.601-0.909 |
| Sex, female | Thrombocytopenia (<50×103/mm3) | 0.700 | 0.543 | 0.682 | 0.540-0.825 |
| Sex, female | Elevated CRP (>3 mg/dL) | **0.867** | 0.478 | 0.732 | 0.580-0.883 |
| Sex, female | aPTT prolongation (≥60 sec) | 0.600 | 0.804 | 0.757 | 0.617-0.896 |
| Confusion | Thrombocytopenia (<50×103/mm3) | 0.533 | **0.913** | 0.783 | 0.639-0.928 |
| Confusion | Elevated CRP (>3 mg/dL) | 0.700 | 0.826 | 0.783 | 0.613-0.954 |
| Confusion | aPTT prolongation (≥60 sec) | 0.733 | 0.717 | **0.786** | 0.625-0.948 |
| Thrombocytopenia (<50×103/mm3) | Elevated CRP (>3 mg/dL) | 0.800 | 0.478 | 0.708 | 0.551-0.865 |
| Thrombocytopenia (<50×103/mm3) | aPTT prolongation (≥60 sec) | 0.600 | 0.804 | 0.750 | 0.606-0.894 |
| Elevated CRP (>3 mg/dL) | aPTT prolongation (≥60 sec) | 0.700 | 0.696 | 0.750 | 0.583-0.917 |

aPTT: activated partial thromboplastin time, CRP: C-reactive protein
